# The Limiting Sequence and Appropriate Amino Acid Ratio of Lysine, Methionine, and Threonine for Seven- to Nine-Month-Old Holstein Heifers Fed Corn–Soybean M-Based Diet

**DOI:** 10.3390/ani9100750

**Published:** 2019-09-30

**Authors:** Yuan Li, Yanliang Bi, Qiyu Diao, Minyu Piao, Bing Wang, Fanlin Kong, Fengming Hu, Mengqi Tang, Yu Sun, Yan Tu

**Affiliations:** 1 Feed Research Institute, Chinese Academy of Agricultural Sciences/ Sino-US Joint Lab on Nutrition and Metabolism of Ruminants, Beijing 100081, China; lyqiu0304@163.com (Y.L.); vetbi2008@163.com (Y.B.); diaoqiyu@caas.cn (Q.D.); tmpark729@126.com (M.P.); a895833622@163.com (F.K.); zgdxsjyzd@163.com (F.H.); 2 College of Animal Science and Technology, China Agricultural University, Beijing 100193, China; wangb@cau.edu.cn; 3 College of Animal Science and Veterinary Medicine, Henan Agriculture University, Zhengzhou 450002, China; tmq0819@163.com (M.T.); sunyu95@163.com (Y.S.)

**Keywords:** amino acid pattern, Holstein heifers, lysine, methionine, threonine

## Abstract

**Simple Summary:**

Research on the amino acid nutrition of cattle is limited, particularly research on the amino acid patterns of growing heifer. This lack of research has made it difficult to minimize the costs and reduce nitrogen emission of dairy heifers. Lysine might be the first limiting amino acid for seven- to nine-month-old Holstein heifers that are fed a corn–soybean meal-based diet, followed by methionine and threonine. The appropriate ratio of lysine, methionine, and threonine—calculated based on the nitrogen retention of seven- to nine-month-old Holstein heifers—were 100:32:57. We expect to reduce the input of protein feed and nitrogen emissions for dairy farms by using this ratio.

**Abstract:**

An “Amino acid (AA) partial deletion method” was used in this experiment to study the limiting sequences and appropriate ratio of lysine (Lys), methionine (Met), and threonine (Thr) in the diets of 7- to 9-month-old Holstein heifers. The experiment was conducted for three months with 72 Holstein heifers (age = 22 ± 0.5 weeks old; BW = 200 ± 9.0 kg; mean ± standard deviation). Following an initial two weeks adaptation period, heifers were allocated to one of four treatments: a theoretically balanced amino acid diet (positive control [PC]; 1.00% Lys, 0.33% Met, and 0.72% Thr), a 30% Lys deleted diet (partially deleted Lys [PD–Lys]; 0.66% Lys, 0.33% Met, and 0.72% Thr), a 30% Met deleted diet (partially deleted Met, [PD–Met]; 1.00% Lys, 0.22% Met, and 0.72% Thr), and a 30% Thr deleted diet (partially deleted Thr [PD–Thr]; 1.00% Lys, 0.33% Met, and 0.45% Thr). Experimental animals were fed a corn–soybean meal-based concentrate and alfalfa hay. In addition, the animals were provided with supplemental Lys, Met, and Thr (ruminal bypass). The results found no differences in the growth performance and nitrogen retention between PD–Thr treatment and PC treatment (*p* > 0.05). The average daily gain (*p* = 0.0013) and feed conversion efficiency (*p* = 0.0057) of eight- to ninr-month-old heifers were lower in both PD–Lys and PD–Met treatment than those in PC treatment. According to growth performance, Lys was the first limiting AA, followed by Met and Thr. Moreover, nine-month-old Holstein heifers in PD–Lys treatment and PD–Met treatment had higher levels of serum urea nitrogen (*p* = 0.0021), urea nitrogen (*p* = 0.0011) and total excreted N (*p* = 0.0324) than those in PC treatment, which showed that nitrogen retention significantly decreased (*p* = 0.0048) as dietary Lys and Met levels decreased. The limiting sequence based on nitrogen retention was the same as that based on growth performance. The appropriate ratio of Lys, Met, and Thr in the diet based on nitrogen retention was 100:32:57. In summary, the limiting sequence and appropriate amino acid ratio of Lys, Met, and Thr for seven- to nine-month-old Holstein heifers fed a corn–soybean meal-based diet were Lys > Met > Thr and 100:32:57, respectively.

## 1. Introduction

Nitrogen (N) loss is a major source of environmental pollution and causes significant economic losses for dairy farms. Given the high amount of N excretion that occurs in dairy cattle relative to their N intake, it is likely that these heifers were fed unbalanced amino acids and that their amino acid requirements were ignored [1]. A key factor for improving dietary amino acid (AA) utilization is the formulation of diets with appropriate amino acid patterns that meet but do not exceed the requirements. Many attempts have been made to decrease the environmental effects of cattle N excretion by manipulating the metabolizable amino acid levels of rations to increase the capture of dietary N by cattle [2,3]. However, deleterious effects may occur in cattle not only due to over-doses amino acid but also due to amino acid imbalances, where there is a lack of an appropriate amino acid pattern. 

The amino acid partial deletion method is the most common method used to develop balanced AA models in animals [4]. This method can be used to determine the sequences needed to limit AA and calculate the optimal ratios. Dorigam et al. [4] estimated the essential AA profile of poultry and determined the ideal pattern for maintenance using this method. Wang et al. [5] also used the deletion method to determine the AA patterns in calf diets. Lysine (Lys), methionine (Met), and threonine (Thr) were found to be the most limiting amino acids, and their concentrations were related to the growth, physiology, and reproductive performance of calves. Ragland et al. [6] also reported that the limiting amino acids for beef cattle were ranked as Lys > Met > Thr, leading us to the conclusion that Lys, Met, and Thr may be the first three limiting amino acids for dairy heifers. 

Research on the amino acid patterns of cattle is limited, particularly regarding the amino acid patterns for each growth stage. Several studies [7] have reported on the amino acid patterns in calf diets [8]. However, one "ideal amino acid pattern" cannot, alone, reliably meet the AA requirements at all growth stages. Balanced AA models should account for changes in growth, body protein composition, and physiological requirements throughout life. The costs of growing heifers are the second largest part in the annual operating expenses of a dairy farm. The lack of optimal amino acid patterns made it difficult to minimize the costs and reduce the nitrogen emissions of heifers. The objective of this study was to determine the amino acid limiting sequence and establish an amino acid ratio in corn–soybean meal and alfalfa hay-based diets for Holstein heifers, aged seven to nine months, using the amino acid partial deletion method.

## 2. Materials and Methods 

### 2.1. Animals, Diets, and Experimental Design 

The experimental procedures were approved by the Animal Ethics Committee of the CAAS. Human animal care and handing procedures were followed throughout the experiment (AEC-CAAS-2017-01).

In this experiment, an AA partial deletion method developed by Wang et al. (1989) [9] was used to prepare the different patterns of the Lys, Met, and Thr diets. The AA levels of the total mixed ration (TMR) in the theoretically balanced AA ration were calculated according to the formula proposed by Zinn et al. (1998) [10]: METR = 1.956 + 0.0292 × ADG × [268 – (29.4 × 0.0557 × BW^0.75^ × ADG^1.097^)/ADG] + 0.112 × BW^0.75^ (METR = methionine requirement; ADG = average daily gain; BW = body weight; BW^0.75^ = metabolic weight). Because of the absence of amino acid patterns in cattle at this stage, Lys and Thr were added according to the AA patterns of the growing swine [11] using a Lys: Met: Thr ratio of 100:30:65. Seventy-two Holstein heifers (age = 5.5 ± 0.5 months old; BW = 200 ± 9.0 kg; mean ± standard deviation) were reared at the Third Dairy Farm of Yinxiang Group Company in Shandong Province, China. The basal diet nutrient level is shown in Table 1.

A completely randomized design was used for this study. Heifers were randomly allocated to four treatments with 18 heifers each, based on body weight and age, and fed one of the four total mixed rations (TMRs): (1) theoretically balanced AA TMR (Positive control, PC); (2) 30% Lys deleted TMR (partially deleted Lys; PD–Lys); (3) 30% Met deleted TMR (partially deleted Met; PD–Met); and (4) 30% Thr deleted TMR (partially deleted Thr; PD–Thr). Ruminal bypass Lys (Yahe Nutrition Co., Beijing, China, 36% content, 80% bypass rate), Ruminal bypass Met (Adisseo Co., Hebei, China, 44.4% content, 50% bypass rate), and Ruminal bypass Thr (King Technology Co., Hangzhou, China, 40% content, 90% bypass rate) were added to the basal TMR diet. The AA levels in the four treatments are shown in Table 2. The amounts of AA added were adjusted monthly according to BW and dry matter intake (DMI). After an adaptation period of two weeks, each animal was weighed and began the study with an average initial BW of 226 ± 10 kg and age of 6 ± 0.5 months old. 

A digestibility and metabolism trial was conducted by selecting four heifers from each treatment during the week before the end of the trial, with a 4-day adaptation period and a 3-day feces and urine collection period. Feces (weight) and urine (volume) outputs were recorded and sampled daily at 07:00, and nitrogen was immediately fixed with 10 mL 10% dilute hydrochloric acid per 100 g feces to determine the N retention (NR). Heifers were housed in individual iron cages (3 × 2.2 m, 6.6 m^2^/ head) bedded with rice husks and fermented cow dung. Fresh water was added ad libitum and replaced daily. The animals were fed TMR twice daily at 08:00 and 17:00. Amino acids were supplemented into the TMR during morning feeding. Individual intakes of TMR were recorded daily and collected weekly during the entire experiment to calculate the dry matter intake (DMI). Environmental conditions (including air temperature) were continuously recorded. The mean air temperature was 11.87 ± 7.54 °C.

The experimental feeding periods were 90 days in duration (September to November 2017). All heifers were immunized according to the standard immunization procedure of the farm, with the brucellosis vaccination administered at 7 months of age. 

### 2.2. Sampling and Analyses

BW and body size were measured before the morning feeding period every 30 days. Diet samples were collected weekly before the morning feeding and stored at −20 °C for further analysis. TMR, feces, and urine samples were sent to the Lab of Ruminant Physiology and Nutrition, Feed Research Institute, Chinese Academy of Agricultural Sciences (Beijing, China) for nutrient analysis. The TMR and feces samples were dried in a forced-air oven at 65 °C for 48 h. Then, the DM (105 °C for 5 h), crude protein (CP), ash, and ether extract (EE) contents were analyzed (method 968.08; AOAC, 1990) [12]. Calcium (Ca) content was analyzed using an atomic absorption spectrophotometer (M9W–700; Perkin–Elmer Corp., Norwalk, CT, U.S.A.) (method 968.08; AOAC, 1990) [12]. Total phosphorus (P) content was analyzed by the molybdovanadate colorimetric method (method 965.17; AOAC, 1990) [12] using a spectrophotometer (UV–6100; Mapada Instruments Co., Ltd., Shanghai, China). The neutral detergent fiber (NDF) and acid detergent fiber (ADF) contents were determined with an Ankom A200 apparatus (Ankom Technology, Macedon, NY, USA) with heat-stable amylase (Ankom Technology) and sodium sulfite (Fisher Scientific, Waltham, MA, USA) and an expressed inclusive of residual ash [13].

A blood sample was collected from six heifers in each treatment (at 24 and 36 weeks of age) before morning feeding, by a jugular venipuncture, and transferred into vacuum tubes without anticoagulants. Serum was immediately separated from the blood by centrifugation at 3000× g at 4 °C for 10 min and stored at −20 °C until analysis. Serum urea nitrogen (SUN) was analyzed using blood colorimetric commercial kits (DiaSys Diagnostics Systems GmbH, Frankfurt, Germany). 

### 2.3. Amino Acid Partial Partial Deletion Method

**Figure 1:**
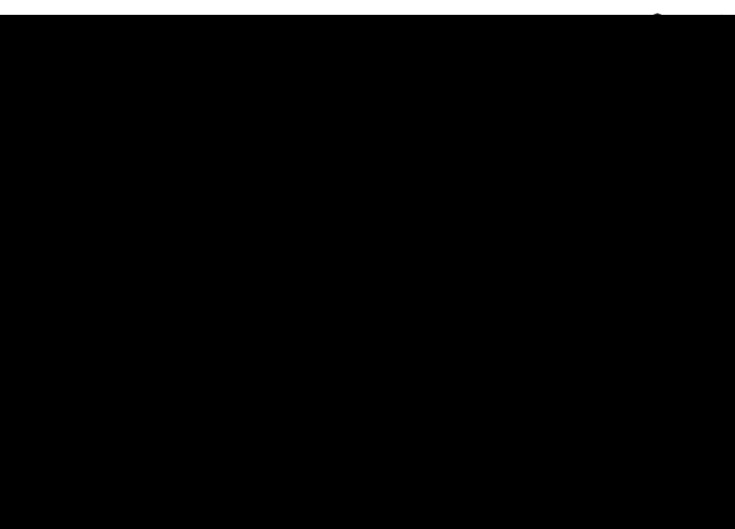
In this model, amino acid intake (AAI) should be presented as the percentage or ratio to control treatment for better distinguishing differences of NR among three AA deleting treatments. To keep the linear relationship between the NR and the first limiting amino acid, the NR should also be converted to the percentage or ratio of the control treatment. The model assumes that deleting the first limiting amino acid (as A) reduces NR to the greatest extent (largest slope); deleting C does not reduce the NR at all (slope = 0), as it remains in excess (over 20%) relative to the first limiting amino acid. Deleting B results in a reduction in the NR intermediate between A and C (0 < Slope B (dashed) < Slope A), and part of B is in excess relative to the first limiting amino acid. In other words, A is the first limiting amino acid while B is second limiting amino acid. According to the principle of the “wooden barrel”, all essential AAs can be controlled by the same limitation by adjusting the amount of AAs in the diet. In this model, B is 10% more than A, which means that we should reduce 10% of B from the control treatment to achieve the minimum addition and ensure it is co-limiting with A [9]. Then, we can calculate the ratio of A and B.

The principle of the amino acid partial partial deletion method is that there is a linear relationship between the first limiting amino acid and the NR. In other words, the NR will decrease greatest after deleting the first limiting animo acid and will result in the largest slope. The model diagram is as follows (Figure 1): 

### 2.4. Statistical Analyses

Data on SUN and N retention were analyzed with a one–way ANOVA procedure using the SAS software (SAS version 9.4; SAS Institute Inc., Cary, NC, USA). Least square means were calculated and separated using the PDIFF option, and differences between diets were detected by Duncan’s multiple comparison in SAS. A MIXED procedure was used to analyze the growth performance data. Month, treatment, and treatment by month of age interactions were fixed effects, and the heifers within each treatment were random effects. The effect of the month was included as a repeated measure. For the repeated measures analysis, the covariance structure with the lowest Akaike information criterion was used. The results were reported as the least squares. A significance level was declared at *p* < 0.05. 

## 3. Results

### 3.1. Growth Performance

The results of the growth performance are presented in Table 3. No significant differences (P > 0.05) were observed in the BW and DMI of heifers among the four treatments during the experiment. However, the ADG (*p* = 0.0013) and G/F (*p* = 0.0057) of heifers in the PD–Lys and PD–Met treatment were decreased significantly compared to PC treatment at eight to nine months old. 

### 3.2. Serum Urea Nitrogen Levels

**Figure 2:**
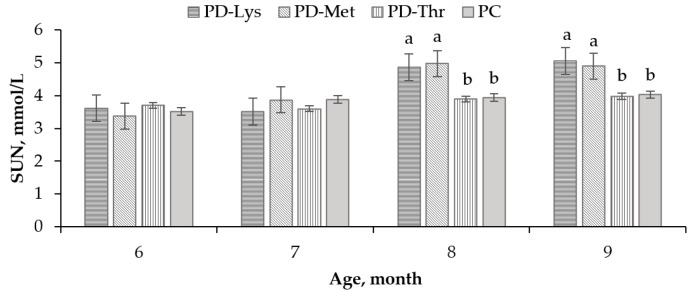
Comparison of serum urea nitrogen levels of seven- to nine-month-old heifers fed corn–soybean based TMRs among the four treatments (n = 24); PD–Lys = 30% Lys deleted TMR diet (diagonal stripes bar), PD–Met = 30% Met deleted TMR diet (vertical stripes bar), PD–Thr = 30% Thr deleted TMR diet (horizonal stripes bar), PC = theoretically balanced amino acid TMR diet (gray bar); The y-axis represents the serum urea nitrogen levels of four treatments; the x-axis was the age of heifers. Error bars indicate SEM. The a,b above the bars indicate the significant differences among treatments (*p* < 0.05).

The SUN levels of heifers aged eight months old (*p* = 0.0013) and nine months old (*p* = 0.0021) in the PD–Lys and PD–Met treatments were higher than those in the PC treatment (Figure 2). No significant differences of SUN were observed between the PD–Thr treatment and PC treatments (*p* > 0.05).

### 3.3. Nitrogen Metabolism

There was no difference in N intake among treatments (Table 4). Total excreted N significantly increased (*p* = 0.0208) when dietary Lys and Met were reduced, as there were significant increases in urine N (*p* = 0.0011). However, fecal N and Digestible N did not differ among four treatments (*p* > 0.05). Moreover, the amount of urine N and NR of heifers in the PD–Thr treatment were not significantly different from those in the PC treatment (*p* > 0.05). 

### 3.4. Appropriate Amino Acid Model

#### 3.4.1. N Retention and Amino Acid Intake 

N retention (NR) and amino acid intake (AAI) based on metabolic weight (Table 5) were converted in proportion to the PC treatment based on the requirements of the “Amino acid partial deletion method model”. Then, the proportions of the intakes of Lys, Met, and Thr in the PD–Lys, PD–Met, and PD–Thr treatment to those in the PC treatment were calculated (e.g., the AAI of Lys in the PD–Lys treatment is 0.60, the AAI of Lys in the PC treatment is 0.90, and the ratio of Lys in the PD–Lys treatment to Lys in the PC treatment is 0.60/0.90 = 0.67 ). After conversion, the ratio of Lys, Met, Thr in amino acid to PC treatment were 0.67, 0.69, and 0.62, respectively. This result differs slightly from 0.7 due to the deferences of the metabolic body weights of the heifers in the four treatments.

#### 3.4.2. Calculation and Model Diagram of the Effect on Nitrogen Retention

The proportions of the three essential AAs were calculated using the simple linear model based on the amino acid partial deletion method [9]. The model diagram of the effect on NR after deleting 30% of Lys, 30% of Met, and 30% of Thr in corn–soybean based diets is shown in Figure 2. 

**Figure 3:**
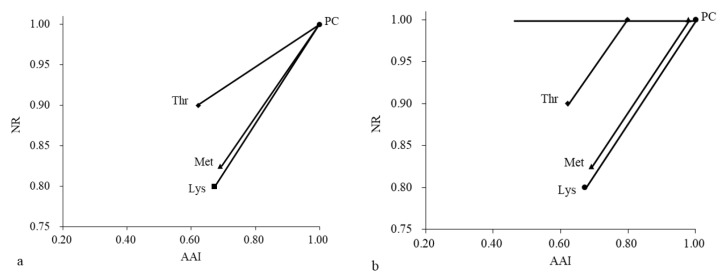
The pattern diagram (**b**) when Met and Thr are converted to an equivalent slope (**a**) with Lys. The y-axis represents the ratio of NR after deleting Lys, Met, Thr to that of the PC treatment; the x-axis is the ratio of the amino acid intake (AAI) in the amino acid deleting treatments to that in the PC treatment. (Lys, ■) = Lys intake and NR level in PD–Lys treatment, (Met, ▲) = Met intake and NR level in PD–Met treatment, (Thr, ◆) = Met intake and NR level in PD–Thr treatment, and (PC, ●) = Lys, Met, Thr intake and NR level in PC treatment; all values =1.

Figure 3a shows the rate of NR in relation to the daily AA intake. The values of AAI (x-axis) and NR (y-axis) are provided in Table 6. Point “PC” represents the corresponding AAIs and NRs of the three AAs in the PC treatment (all values = 1 only one point). “Lys” is the point of the Lys intake and NR in the PD–Lys treatment (0.67, 0.80). “Met” is the point of the Met intake and NR in the PD–Met treatment (0.69, 0.83). “Thr” is the point of Thr intake and NR in the PD–Thr treatment (0.62, 0.85). 

The slope (Table 5) describes the effect of deleting an AA from the PC on the NR (e.g., for Lys, (1–0.80)/(1–0.67) = 0.61). Among the three AAs, a higher slope for the Lys deletion treatment (PD–Lys) means that Lys is the first limiting AA in the PC treatment. The limiting sequence of the three amino acids is ranked as: Lys > Met > Thr.

Figure 3b is the pattern diagram for when Met and Thr are converted to an equivalent slope with Lys. To calculate the proportion of each AA that could be removed from the PC amino acid pattern to make it equally limiting to Lys, it was assumed that when all AAs are equally limiting, they should all have the same slope. Therefore, the required amount of Met can be caculated as: S (Lys) = (1 − 083)/(x − 0.69)—that is, 0.61 = (1 − 0.83)/(x − 0.69), x = 0.98, thence 1.00 − 0.98 = 0.02. In other words, 0.02 of Met should be removed from the PC to make the Met co-limiting with the Lys (the actual requirement of Met is 0.98 × Met in the PC treatment). In the same way, we calculate that the 0.21 of Thr should be removed from the PC treatment when it is equally limited with Lys (the actual requirement of Thr is 0.79 × Thr in the PC treatment). 

#### 3.4.3. Appropriate Amino Acid Ratio

An appropriate amino acid model of seven- to nine-month-old Holstein heifers is shown in Table 6 (calculated from Figure 2). S (slope) represents the effect of deleting 30% amino acid on N retention (S = (1 − NR)/(1 − AAI), calculated from Figure 2a). The S value of the Lys deleted treatment was the highest, indicating that Lys was the first limiting amino acid. P (proportion) is the proportion of amino acid (except for Lys) in the PC treatment when it was equally limited to that of Lys (P = [(1−NR) + S × AAI]/S, calculated from Figure 2b). The P value was calculated based on the principle of “equal limitation means equal slope.” C (concentration) is the actual concentration of amino acid when it was equally limited with Lys (C = AAI ( in PC treatment) × P). R (ratio) is the ratio of the actual amino acid concentration to the Lys concentration (R = AA/Lys). The optimal ratios based on the NR of the three amino acids for seven- to nine-month-old Holstein heifers was 100:32:57.

## 4. Discussion

### 4.1. Growth Performance and Body Size

The function of dietary protein is determined by amino acid composition, the nutrient digestive abilities of animals, and how well the composition of absorbed amino acid matches the balance required by the animals. The deficiency and overdose of certain amino acids in the diet will cause an imbalance between amino acids and thus affect the growth and development of the animals. For calves, the addition of Lys and Met in a milk replacer significantly increased the feed conversion efficiency of calves, but the addition of Thr had no significant effect on the growth performance of calves [7]. Ludden et al. [14] observed that supplementation with Lys improved the ADG in the growing cattle. Awawdeh et al. [15] showed that when Met was limiting amino acid, the dietary supplementation of other amino acids increased the utilization efficiency of Met and increase the growth of bulls. In this experiment, deleting 30% of Lys and Met led to a decrease of ADG and G/F. Such a reduction of growth performance might be due to the unbalanced amino acids. Another important observation is that DMI seems not to be affected by treatment. Wang et al. [16] found no significant differences in the DM and N intake of dairy cows after adding Lys and Met to the diet. That is to say, growth performance is affected by limiting amino acid deficiencies rather than feed intake [17]. Lys and Met might be the first two limiting amino acids for growing cattle. Unlike Lys and Met, the growth responses to Thr deletion were not significantly decreased. It remains possible that the theoretical Thr addition in this experiment was relatively higher than the requirement of heifers due to the absence of accurate data on Thr requirements in this trial.

### 4.2. Serum Urea Nitrogen and Nitrogen Retention

SUN, as an end metabolite of the liver’s N metabolism [18], is negatively correlated with the utilization rate of protein [19]. The balance of amino acids is the basic condition needed to improve protein utilization and reduce SUN concentration [19]. Jiang et al. [20] estimated sharp decreases in the content of SUN and the emission of urine nitrogen, as well as an increase of the N retention of cows after adding Met and Lys in their diets. In our study, SUN concentration increased after deleting 30% of dietary Lys and Met, which might indicate an imbalance of amino acids and decrease N utilization. Urine N is the main excretion pathway of SUN, accounting for a large part of the N excretion of heifers. An amino acid balanced diet can improve the N utilization rate and reduce the excretion of fecal and urine nitrogen (about 46%) of dairy cows, especially urine N excretion [21]. Adding Lys and Met to diets can promote a balance of amino acids, reduce urinary nitrogen concentration, and improve the protein utilization rate of dairy cows [22]. Recent research by Lee et al. [2] concluded that the efficiency of feed N absorbed by the small intestine increased when dietary amino acid was balanced. We also observed that urine N was significantly increased as the Lys and Met levels decreased. Therefore, it was further confirmed in this study that deleting Lys and Met led to an imbalance of amino acids, which resulted in an increase of urine nitrogen. 

N retention reflects the efficiency of protein deposition and amino acid utilization [23], which is also closely related to the production performance of animals [24]. A balance of amino acids in the diet can enhance the digestion and absorption of N in animals [25]. In particular, the metabolic amount of the first limiting amino acid has a linear relationship with N retention [26]. Balancing a complete amino acid profile increased the efficiency of dietary N utilization in both a low and a high small intestine protein supply [27]. The efficiencies of nitrogen estimated in the current study confirmed that adding Lys to the Lys deficient diet of calves reduced the rate of N excretion and increased the rate of N deposition [28]. Conversely, heifers fed with Lys and Met deficient diets caused an increase of nitrogen retention, indicating inefficiencies in their use of absorbed amino acid for protein accretion [25]. Importantly, the effects of dietary Lys, Met, and Thr levels on the N retention of heifers is not consistent and largely depends on the balance and limiting sequences of these three amino acids. In this study, the decrease of N retention, in combination with the deficiency of Lys and Met, indicated that Lys and Met are the first and second limiting amino acids for heifers , respectively.

In addition, an increase in N retention was commonly reported for cows fed diets with a supplementation of rumen-protected amino acids [29,30], similar to the present study, which indicated that the added ruminal protected amino acids were effectively protected from ruminal degradation and guaranteed amino acids to be released and absorbed in the small intestine for better utilization. Dietary supplementation of rumen-protected Met and Lys could ensure a balance of amino acids, promote the increase of nitrogen deposition, and improve the utilization rate of proteins [31]. In this case, supplementation with rumen-protected amino acids may be a successful strategy for establishing the amino acid pattern of heifers based on dietary amino acids. 

### 4.3. Limiting Sequence and Appropriate Ratio of Amino Acid

The limiting sequence of amino acids in ruminants was affected by the composition of their diets. Maize silage/maize gain based diets can supply adequate protein but do not provide enough Lys to growing cattle, which indicates that Lys is the first limiting amino acid [32]. Klemesrud et al. [33] also reported that Lys is the first limiting amino acid in steers fed with diets containing large amount of maize products. Wang [8] found that Lys was the first limiting amino acid (the second and third were Met and Thr, respectively) due to the large decrease of ADG in calves fed with a milk replacer, starters, and Leymus chinensis after reducing Lys. We found that the limiting sequence of seven- to nine-month-old Holstein heifers was Lys > Met > Thr, based on corn–soybean meal–alfalfa TMRs. Therefore, Lys plays the most important role in the growth of heifers that are fed rations made from corn–soybean meal.

An optimal amino acid pattern is needed as a standard for evaluating the diets of animals. The requirements of amino acids are not well defined for heifers with corn–soybean meal-based diets, and modifying amino acid patterns can increase the bypass protein efficiency [34]. When the Lys ratio is expressed, variation in the estimated requirement of the specific AA is greatly reduced compared to the amino acid ratio of the diets [25]. NRC (2012) [35] pointed out that the ideal amino acid pattern should be expressed as the ratio of amino acid to Lys. In this study, Lys happened to be the first limiting amino acid, so the calculated model is appropriate. The results from this study show that the appropriate pattern of amino acids in the diet based on maximum N retention in seven- to nine-month-old Holstein heifers (fed with corn–soybean meal) was 100:32:57. However, we did not precisely determine the limiting amino acid pattern because of our inability to accurately calculate metabolic proteins, so this pattern cannot be applied to all type of diets. This may offer an explanation for the differences between this pattern and the patterns for calves and cows (Table 7). Of course, the amino acid requirement for growing heifers might change according to age. It is possible that differences in diet type and digestion among heifers, calves, and cows directly affect the profile of delivered amino acids to the intestine. However, the ruminal bypass amino acid products and microbial metabolism made it difficult to determine whether there was a large difference between intake N and metabolic N. Therefore, further research is needed to determine the precise amino acid patterns based on metabolic protein. 

Amino acid is mainly used for the bodily growth and development of heifers. Amino acid requirements are mainly determined by body protein retention and N emission, similar to beef cattle, so the amino acid requirement of heifers may be determined by the composition of the body’s amino acid [36]. However, the amino acid pattern in this research was different from that of beef cattle (Table 7). Studies have shown dietary amino acid patterns are not equivalent to carcass amino acid composition. Decomposition and conversion by intestinal bacteria produced a significant difference between amino acid in the diet and amino acid absorbed into the blood. Moreover, different tissues have different uses and metabolic efficiencies for amino acid, which may cause a deviation between a carcass’s amino acid composition and dietary amino acid patterns. Considering animal welfare and economic benefits, the calculated amino acid model was not verified by a carcass’s amino acid components. Whether a carcass’s amino acid components can be used as an appropriate amino acid model for growing heifers needs to be further verified.

## 5. Conclusions

In this study, there were negative effects on the average daily gain, feed conversion rate, and nitrogen retention of seven- to nine-month-old heifers after deleting 30% dietary Lys and Met. However, deleting Thr content did not affect the growth performance and N metabolism of heifers. The sequence of the three amino acids for seven- to nine-month-old Holstein heifers that were fed a TMR of corn–soybean meal concentrate and alfalfa hay was Lys > Met > Thr. Additionally, the appropriate amino acid ratio calculated from nitrogen retention of this ratio of diet was 100:32:57.

## Figures and Tables

**Table 1:** Composition and nutrient levels of basal total mixed ration (TMR) (dry matter basis).

Ingredients	Contents, %	Nutrient Levels ^2^	Levels
Corn	45.67	Metabolizable energy, (MJ/kg)	10.13
Soybean meal	11.97	Crude protein, %	14.95
Wheat bran	15	Ether extract, %	3.04
Alfalfa hay	25	Ash, %	7.58
Limestone	1.06	Neutral detergent fiber, %	29.22
Salt	0.3	Acid detergent fiber, %	13.99
Premix ^1^	1	Calcium, %	1.12
Total	100	Phosphorus, %	0.60
		Lysine, %	0.51
		Methionine, %	0.07
		Threonine, %	0.49

^1^ The premix provided the following minerals and vitamins for TMR: Cu, 12.5mg/kg; Fe, 90 mg/kg; Zn, 90 mg/kg; Mn, 30 mg/kg; I, 1.0 mg/kg; Se, 0.3 mg/kg; Co, 0.5 mg/kg; vitamin A, 15,000 IU/kg; vitamin D35,000 IU/kg; vitamin E, 50 mg/kg; ^2^ nutrient levels were measured values, except for metabolizable energy, which was measured and calculated through digestibility and metabolism trials. The energy of CH_4_ was calculated by equation 10.21 (IPCC, 2006), Ym = 5.5%.

**Table 2:** Amino acid (AA) levels of TMRs (dry matter basis).

Items	Treatments ^1^ (%)			
	PC	PD–Lys	PD–Met	PD–Thr
Total AA content				
Lysine	1.00	0.66	1.00	1.00
Methionine	0.33	0.33	0.22	0.33
Threonine	0.72	0.72	0.72	0.45
AA content in basal diet				
Lysine	0.51	0.51	0.51	0.51
Methionine	0.07	0.07	0.07	0.07
Threonine	0.45	0.45	0.45	0.45
Exogenously added AA				
Lysine	0.49	0.15	0.49	0.49
Methionine	0.25	0.25	0.15	0.25
Threonine	0.23	0.23	0.23	0.00

^1^ Treatments: PC = theoretical amino acid balance TMR; PD–Lys = 30% Lys deleted TMR; PD–Met = 30% Met deleted TMR; PD–Thr = 30% Thr deleted TMR.

**Table 3:** Effects of deleting Lysine (Lys), Methionine (Met), and Threonine (Thr) levels in corn–soybean based TMR on the growth performance of heifers aged seven to nine months old (n = 72).

Items ^1^	Treatments ^2^				SEM	*p* Value ^3^		
	PD–Lys	PD–Met	PD–Thr	PC		T	M	T × M
BW, kg								
Average	273.7	273.9	276.3	274.8	2.98	0.4798	<0.0001	<0.0001
6 mon	227.5	227.0	229.9	228.5	4.08	0.4647		
7 mon	258.0	257.8	259.3	257.6	4.00	0.7016		
8 mon	282.7	284.3	282.5	280.3	4.52	0.3667		
9 mon	326.4	326.8	333.4	333.0	4.02	0.0997		
ADG, kg								
Average	1.04	1.09	1.09	1.11	0.029	0.1566	<0.0001	<0.0001
6–7 mon	0.98	1.02	0.94	0.95	0.075	0.2848		
7–8 mon	0.95	0.95	0.90	0.89	0.079	0.4946		
8–9 mon	1.21 ^c^	1.30 ^bc^	1.44 ^ab^	1.48 ^a^	0.080	0.0013		
DMI, kg								
Average	7.15	7.16	7.16	7.05	0.050	0.1073	<0.0001	<0.0001
6–7 mon	6.25	6.20	6.26	6.28	0.091	0.2942		
7–8 mon	6.99	6.98	6.94	6.84	0.089	0.0916		
8–9 mon	8.21	8.28	8.16	8.25	0.088	0.2297		
Feed conversion rate, G/F								
Average	0.146	0.152	0.152	0.156	0.008	0.1452	0.0001	0.0002
6–7 mon	0.156	0.164	0.151	0.155	0.011	0.2138		
7–8 mon	0.135	0.134	0.127	0.130	0.010	0.5009		
8–9 mon	0.147 ^c^	0.158 ^bc^	0.176 ^ab^	0.181 ^a^	0.012	0.0057		

^1^ BW=body weight, ADG=average daily gain, DMI=dry matter intake; ^2^ Treatments: PC = theoretical amino acid balance TMR; PD–Lys = 30% Lys deleted TMR; PD–Met = 30% Met deleted TMR; PD–Thr h = 30% Thr deleted TMR; ^3^ T = Treatment, M = Month of age, T × M = The interaction between treatment and month of age; ^a,b,c^ values within the same row with different superscripts are different (*p* < 0.05).

**Table 4:** Effects of deleting Lysine, Methionine, and Threonine levels in corn–soybean based TMRs on nitrogen metabolism of heifers aged seven to nine months old (n = 16).

Items ^1^	Treatments ^2^				SEM	*p* Value
	PD–Lys	PD–Met	PD–Thr	PC		
Intake N, g·(kg^−1^ BW^0.75^) d^−1^	2.92	2.97	2.90	2.87	0.020	0.2961
Fecal N, g·(kg^−1^ BW^0.75^)·d^−1^	0.90	0.82	0.80	0.79	0.015	0.1223
Urine N, g·(kg^−1^ BW^0.75^)·d^−1^	1.06 ^b^	1.16 ^a^	1.02 ^b^	0.88 ^c^	0.033	0.0011
Total excrete N, g·(kg^−1^ BW^0.75^)·d^−1^	1.96 ^b^	1.98 ^a^	1.82 ^ab^	1.67 ^ab^	0.032	0.0208
N retention, g·(kg^−1^ BW^0.75^)·d^−1^	0.96 ^b^	0.99 ^b^	1.08 ^ab^	1.20 ^a^	0.034	0.0324
Digestible N, g·(kg^−1^ BW^0.75^)·d^−1^	2.02	2.15	2.06	2.08	0.020	0.2908
N utilization, %	33.08 ^b^	33.26 ^b^	34.96 ^b^	41.77 ^a^	1.210	0.0048
N digestibility, %	69.50	72.38	70.79	72.6	0.512	0.0798

^1^ N = nitrogen; Total excrete N=Fecal N + Urine N, Absorbed N =Intake – Total excrete N, NR (N retention) = N intake – fecal N – urinary N, N utilization = (N intake – fecal N excretion)/N intake × 100%, N digestibility = (N intake – fecal N excretion)/N intake × 100%; ^2^ treatments: PC = theoretical amino acid balance TMR; PD–Lys = 30% Lys deleted TMR; PD–Met = 30% Met deleted TMR; PD–Thr = 30% Thr deleted TMR; ^a, b, c^ values within the same row with different superscripts differ (*p* < 0.05).

**Table 5:** The proportions of amino acid intake and nitrogen retention in PD–Lys, PD–Met, and PD–Thr to those in the PC treatment.

Items ^1^	Based on Metabolic Body Weight, g·(kg^−1^ BW^0.75^)·d^−1^				The Ratio to PC			
	NR ^2^	AAI ^2^			NR	AAI		
		Lys	Met	Thr		Lys	Met	Thr
PD–Lys	0.96	0.60	0.29	0.65	0.80	0.67	1.00	1.00
PD–Met	0.99	0.90	0.20	0.65	0.83	1.00	0.69	1.00
PD–Thr	1.02	0.90	0.29	0.40	0.85	1.00	1.00	0.62
PC	1.20	0.90	0.29	0.65	1.00	1.00	1.00	1.00

^1^ PD–Lys = 30% Lys deleted treatment; PD–Met = 30% Met deleted treatment; PD–Thr = 30% Thr deleted treatment; PC = theoretical amino acid balanced treatment; ^2^ NR=N retention; AAI=amino acid intake.

**Table 6:** The appropiate amino acid ratio of Lysine (Lys), Methionine (Met), and Threonine (Thr) based on the nitrogen retention (NR) of heifers aged seven to nine months old, fed corn–soybean meal-based TMRs.

Items ^1^	S	P	C	R
Lys	0.61	1.00	69.99	100.00
Met	0.57	0.98	22.36	31.95
Thr	0.26	0.79	39.78	56.84

^1^ S(Slop) = (1-NR)/(1-AAI); P(proportion) = [(1-NR) + S × AAI]/S; C (concentration) = AAI × P; R (ratio) = AA/Lys.

**Table 7:** The amino acid ratio of calves and cows in previous studies.

Stage	Index ^1^	Lys: Met: Thr Ratio	Reference
Calves	NR	100:26:66	Gerrits et al., 1997 [37]
Calves	maximum ADG	100:31:77	Hill et al., 2008 [7]
Calves	maximum ADG	100:35:63	Wang et al., 2011 [8]
Beef cattle	body amino acids	100:31:61	NRC (2016) [38]

^1^ NR = nitrogen retention; ADG = average daily gain.
